# Serum Lactate Predicts Adverse Outcomes in Emergency Department Patients With and Without Infection

**DOI:** 10.5811/westjem.2016.10.31397

**Published:** 2016-12-07

**Authors:** Kimie Oedorf, Danielle E. Day, Yotam Lior, Victor Novack, Leon D. Sanchez, Richard E. Wolfe, Hans Kirkegaard, Nathan I. Shapiro, Daniel J. Henning

**Affiliations:** * Beth Israel Deaconess Medical Center, Department of Emergency Medicine, Boston, Massachusetts; † Ben-Gurion University of the Negev, Clinical Research Center Soroka University Medical Center, and Faculty of Health Sciences, Beersheba, Israel; ‡ Aarhus University Hospital, Research Center for Emergency Medicine, Aarhus, Denmark

## Abstract

**Introduction:**

Lactate levels are increasingly used to risk stratify emergency department (ED) patients with and without infection. Whether a serum lactate provides similar prognostic value across diseases is not fully elucidated. This study assesses the prognostic value of serum lactate in ED patients with and without infection to both report and compare relative predictive value across etiologies.

**Methods:**

We conducted a prospective, observational study of ED patients displaying abnormal vital signs (AVS) (heart rate ≥130 bpm, respiratory rate ≥24 bpm, shock index ≥1, and/or systolic blood pressure <90 mmHg). The primary outcome, deterioration, was a composite of acute renal failure, non-elective intubation, vasopressor administration or in-hospital mortality.

**Results:**

Of the 1,152 patients with AVS who were screened, 488 patients met the current study criteria: 34% deteriorated and 12.5% died. The deterioration rate was 88/342 (26%, 95% CI: 21 – 30%) for lactate < 2.5 mmol/L, 47/90 (52%, 42 – 63%) for lactate 2.5 – 4.0 mmol/L, and 33/46 (72%, 59 – 85%) for lactate >4.0mmol/L. Trended stratified lactate levels were associated with deterioration for both infected (p<0.01) and non-infected (p<0.01) patients. In the logistic regression models, lactate > 4mmol/L was an independent predictor of deterioration for patients with infection (OR 4.8, 95% CI: 1.7 – 14.1) and without infection (OR 4.4, 1.7 – 11.5).

**Conclusion:**

Lactate levels can risk stratify patients with AVS who have increased risk of adverse outcomes regardless of infection status.

## INTRODUCTION

The use of lactate to identify patients at risk for adverse outcomes and to guide treatment decisions for emergency department (ED) patients with infection has gained widespread adoption based upon a number of studies.^1^–^6^ The Surviving Sepsis Campaign^7^ has incorporated the measurement of serum lactate concentrations into its most current guidelines, emphasizing measurement within three hours of identification of sepsis. Despite there being many causes of elevated lactate levels, lactate functions well as a severity marker in ED patients with infection,^1^,^8^ and it has been widely adopted as a method to risk stratify ED patients with infection.

In non-infectious diseases, such as cardiac arrest, ST-elevation myocardial infarction (STEMI),^9^,^10^ trauma^11^ and other causes of hospitalization,^1^,^12^,^13^ lactate levels have also demonstrated utility for risk stratification. For instance, current trauma guidelines^14^ recommend using lactate to risk stratify patients and guide fluid administration, and a lactate is recommended for the diagnosis and staging of shock in the intensive care unit. 15 Furthermore, a recent study from Denmark demonstrated that stratified lactate concentrations predict 10-day mortality in an undifferentiated acute care population that had a lactate measured.^16^ Although this study did not assess the potential effect of the underlying disease, it further supports the use of lactate to risk stratify patients regardless of diagnosis.

Animal model evidence suggests that sepsis alters regional perfusion, even after adjusting for decreased cardiac output, and that this sepsis-specific perfusion derangement is associated with elevated lactate levels compared with non-septic etiologies.^17^ Furthermore, lactate metabolism is decreased during sepsis, compared to sterile inflammation, leading to prolonged elevation of lactate in septic animals.^18^ Based on the altered physiology of lactate production and clearance during sepsis, it is plausible for lactate concentrations to have different strengths of association with adverse outcomes depending on the underlying disease. Therefore, for clinicians ordering a serum lactate to risk stratify potentially ill patients, there remains a need to understand if the strength of association is disease-dependent, or whether lactate concentrations add the same predictive value in non-infectious conditions. It is possible that the predictive value of serum lactate concentrations is modified by the underlying diagnosis, requiring clinicians to interpret lactate values differently depending on the disease process.

The objectives of this study were the following: 1) to describe the association between lactate concentrations and adverse outcomes in patients with and without infectious causes of abnormal vital signs (AVS); and 2) to assess whether lactate concentrations add significant prognostic value to clinical data when predicting adverse outcomes in a single ED population stratified by infectious or non-infectious cause of illness.

## METHODS

This was a pre-planned secondary analysis of a prospective, observational cohort study of a consecutively enrolled population of ED patients with AVS who also had a lactate level obtained during the routine course of clinical care. We enrolled patients with AVS to target an “at risk” population.^19^ Patients were enrolled from November 11, 2012, to January 31, 2013. The study was conducted at an urban, academic, tertiary care hospital with 55,000 annual ED visits. This study was granted waiver of informed consent after expedited review by the human subjects committee of our institutional review board.

We included patients above 18 years old with the presence of at least one of the following AVS at triage or during their ED stay: heart rate ≥ 130, respiratory rate ≥ 24, shock index ≥1, or systolic blood pressure < 90 mmHg, or a lactate level ≥ 4mmol/L. Vital sign thresholds were chosen based on our hospital system’s previously published criteria to identify patients at higher risk of short-term adverse outcomes^20^ and prior investigations of AVS and elevated shock index.^21^,^22^ Exclusion criteria were the following: patients with tachycardia due to atrial fibrillation with rapid ventricular response or supraventricular tachycardia who were then discharged once rate control was achieved; vital sign abnormalities due to intoxication, withdrawal, psychiatric disorder, seizure, or simple trauma (i.e., fracture). We also excluded patients who were discharged from the ED. Excluding these patients focused our investigation on a population with AVS due to critical illness and needing further risk stratification in the original cohort. For the current study, we also excluded patients without a lactate measured in the ED. We continuously and prospectively screened patients in the ED for possible inclusions using our information technology system. If patients had qualifying vital signs in triage, in nursing notes, or through the bedside monitors, then they were identified for possible inclusion in the study. Identified patients then underwent a confirmatory chart review to affirm the presence of inclusion criteria and absence of exclusion criteria.

We reviewed hospital charts and abstracted the history of present illness, past medical history, pre-hospital and ED administered medications, and vital signs from the emergency physician notes. Past medical history and current medications were abstracted from the admission note from the inpatient team if the ED note was incomplete. Vital signs at the time of inclusion were used. We included the first peripheral venous or central venous lactate level sample, consistent with previous studies based on venous sampling^3^,^5^,^23^. Data abstraction was performed by two research assistants, trained and directly supervised by the principal investigator (PI). Chart abstraction was performed without knowledge of the final diagnosis, since adjudication of diagnosis was performed at a later date. Demographic information, hospital length of stay, and laboratory testing, including first lactate obtained in the ED, were matched to each patient from the hospital’s electronic database after all abstractions were completed.

We defined the primary composite outcome “deterioration” as one or more of the following at any time during the present hospitalization: acute renal failure, non-elective intubation, vasopressors administration, and in-hospital mortality. Acute renal failure was defined as a creatinine value double the patient’s most recent available value or new initiation of hemodialysis during admission. If a prior creatinine measurement was not available, an initially elevated creatinine was marked as acute renal failure if the value decreased greater than 50% during hospitalization. The secondary outcome was in-hospital mortality. We defined “shock in the ED” as 1) systolic blood pressure < 90 mmHg after at least 1L fluid; 2) at least two systolic blood pressure readings < 90mmHg and with clear nursing or physician documentation of withholding fluids due to concern for fluid overload; or 3) use of vasopressors. The variable “triage acuity” (1, 2, or 3 inversely related to severity) was determined by the triage nurse at the time that patients arrived in the ED.

The presence of an infection and outcomes during admission were adjudicated by the PI through a review of both ED and hospital documentation after discharge from the hospital. The diagnosis of infection was guided by objective data (e.g. blood cultures, chest radiograph interpretations, urinalysis, etc.), and the final diagnosis was a clinical judgment based on integration of this data. A second reviewer adjudicated the first 500 subjects enrolled in the primary study to assess inter-rater reliability. This secondary analysis includes 343 patients (70%) that had a second review, and in this subset kappa = 0.85 (95% confidence intervals (CI): 0.78 – 0.90).

### Data Analysis

We performed statistical analysis using SPSS version 18. The primary outcome was deterioration and secondary outcome was in-hospital mortality. The variable of interest was initial blood lactate level, which was stratified as low (< 2.5 mmol/L), intermediate (2.5 – 4.0 mmol/L) or high (> 4.0 mmol/L). To allow for easier clinical interpretation and application we used stratified lactate levels, as opposed to continuous lactate levels,.

Continuous variables were presented as mean ± SD and were compared using Student’s t-test. Variables were compared using chi-square test, Mann-Whitney test, and chi-square test for trend, as appropriate. We tested the association between stratified lactate levels and both deterioration and mortality outcomes grouped by infection status.

We created multivariate logistic regression models to assess whether lactate was independently associated with deterioration and/or mortality. Variable selection for the models was based on clinical and statistical significance, defined as p < 0.05. We used the n/10 rule to determine the maximum number of covariates to include in each model to prevent overfitting. We reported a final model and used the Hosmer-Lemeshow test for assessing model calibration and c-statistics for modeling discriminatory abilities.

Integrated discrimination improvement (IDI) was used to assess the added discriminate value of including stratified lactate to models predicting the outcomes of deterioration and mortality without lactate. IDI compares the predicted probability of an event for models before and after the addition of stratified lactate, and tests the improvement in reclassification of subjects with and without an event (i.e. deterioration). IDI was performed for patients with and without infection and for each outcome, using the best model created without lactate as a reference.^24^

Finally, we used locally weighted polynomial regression (LOESS) to analyze the association between lactate values expressed as a continuous variable and the adjusted probability of each outcome (deterioration or mortality) in both groups.

## RESULTS

### Patient Population

We identified 1,152 patients with AVS, of whom 366 met clinical exclusion criteria. Of the remaining 786 patients eligible for this analysis, 298 did not have ED lactate measurements, leaving 488 for the analysis. The mean age of our population was 63 (± 18) years. There were 168 patients (34.4%) who had a deterioration, and 61 (12.5%) died. Of the 488 patients analyzed, 286 (58.6%) had infectious etiologies; the non-infectious etiologies are shown in supplemental Table 1. The population without infection had a significantly higher prevalence of diabetes (34% vs. 24%, p = 0.02) and congestive heart failure (25% vs. 17%, p = 0.03). A comparison of vital sign variables between groups showed that patients with infection had a higher average heart rate and temperature (Table 1).

Overall, 342/488 (70.1%) had lactate < 2.5 mmol/L, 100/488 (20.5%) had lactate 2.5 – 4.0 mmol/L, and 46/488 (9.4%) had lactate > 4.0 mmol/L. Table 2 shows the distribution of deterioration stratified by lactate level for both infected and non-infected patients. We were unable to detect a difference (p = 0.92) when comparing the distribution of patients with and without infection between the stratified lactate groups.

### Clinical Outcomes

Table 3 depicts the clinical outcomes of the cohort. Our data showed no difference between the two diagnostic groups in mortality rate (p = 0.95) or deterioration (p = 0.76). There was a significantly higher frequency of shock in the ED (p = 0.002) and administration of vasopressors (p<0.001) in patients with infection.

**Figure 1:**
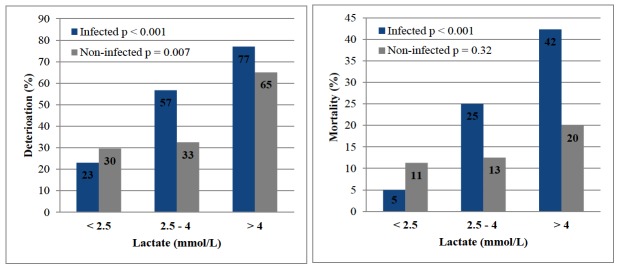
Incidence of deterioration and death in patients with and without infection stratified by lactate concentration; P-values by chi-square test for trend for positive association across stratified lactate levels.

Overall, the deterioration rate was 88/342 (26%) for lactate < 2.5 mmol/L, 47/100 (47%) for lactate 2.5 – 4.0 mmol/L, and 33/46 (72%) for lactate >4.0mmol/L. Mortality was 26/342 (8%) for lactate < 2.5 mmol/L, 20/100 (20%) for lactate 2.5 – 4.0 mmol/L, and 15/46 (33%) for lactate > 4.0 mmol/L. Figure 1 shows the rates of deterioration and mortality by lactate levels for each group. Both groups demonstrated a significant positive association between stratified lactate level and deterioration rates (p<0.001 for infected and p = 0.007 for non-infected patients). Our data likewise showed lactate levels were associated with mortality in patients with infection (p<0.001), but not patients without infection (p = 0.32).

### Discrimination Analysis

Patients with infection: The model for predicting deterioration in patients with infection is shown in Table 4a. Using non-lactate covariates resulted in an initial model with c-statistic of 0.81 (95% CI: 0.76–0.86) (p <0.001) when predicting deterioration. When lactate > 4.0 mmol/L is added to the reference model, area under the curve (AUC) = 0.83 (95% CI: 0.78–0.88) (p <0.001), with an absolute IDI of 0.03 (95% CI: 0.00–0.05) (p<0.001), showing a significant improvement in prediction. The model for predicting mortality in patients with infection is shown in Table 4b. The model using non-lactate covariates predicting mortality had a c-statistic of 0.80 (95%CI: 0.74–0.86) (p <0.001). This improves to 0.83 (95% CI: 0.78–0.88)(p <0.001) when lactate > 4.0 mmol/L is added to the model, with an absolute IDI of 0.02 (95%CI: 0.00–0.05) (p<0.001) for this model.

Patients without infection: The analysis to predict deterioration among patients without infection is seen in Table 4c. The best model predicting deterioration without using lactate had a c-statistic 0.76 (95% CI: 0.69–0.83) (p <0.001). Adding lactate > 4mmol/L to this model yielded an AUC = 0.78 (95% CI: 0.71–0.85) (p <0.001). The new model had an absolute IDI of 0.03 (95% CI: 0.00–0.06) (p<0.04) suggesting that addition of lactate level improved the discriminatory value of the model for predicting deterioration.

Table 4d shows the model for predicting mortality among patients without infection. The multivariate regression model without lactate > 4.0 mmol/L achieved an AUC of 0.62 (95% CI: 0.54–0.70), and after adding lactate to the model, had an AUC = 0.62 (95% CI: 0.54–0.70). The absolute IDI was 0.00 (95% CI : 0.00–0.02) (p = 0.07). Of note, lactate > 4.0 mmol/L was not significant in this model (p = 0.81).

**Figure 2a, b:**
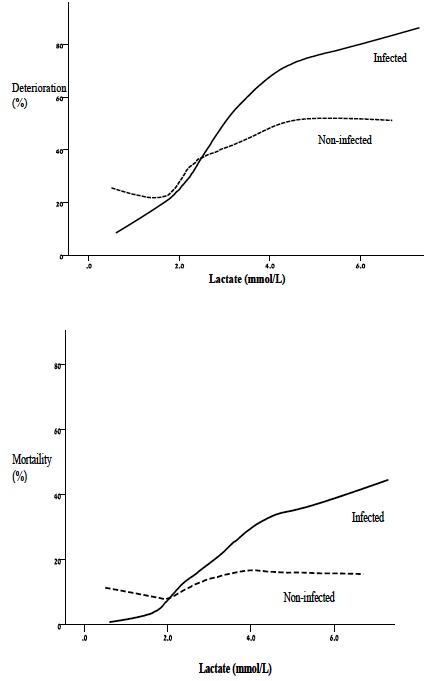
Both groups exhibit increasing deterioration as lactate levels increase, although this figure suggests that the response may be larger in patients with infection. *LOESS*, locally weighted polynomial regression.

The LOESS graphs for adjusted outcomes and lactate levels provide a visual representation of the dose-response association for both deterioration and mortality between patient groups (Figure 2a+b).

## DISCUSSION

This analysis evaluates the relationship between lactate concentrations and patient outcomes for patients with infectious and non-infectious causes of AVS. In patients with infection, a statistically significant association exists between both deterioration and mortality and an increasing lactate level. The regression models for predicting deterioration and mortality in infected patients further demonstrate that lactate concentrations add value to the prediction of both outcomes. Likewise, lactate concentrations can also assist in predicting deterioration in patients without infection. In this non-infected group, increasing lactate predicted increasing rates of deterioration. The model for non-infected patients likewise suggests that lactate levels can predict deterioration. While neither the stratified analysis nor regression model for patients without infection demonstrated a significant relationship between lactate concentration and the outcome of mortality, this result is possibly due to type II error since the study was not powered to evaluate mortality primarily.

As mentioned before, the physiology of sepsis likely causes increased lactate production^17^ and decreases lactate metabolism,^18^ which could alter the relationship between lactate concentrations and adverse outcomes seen in patients with and without infection. The LOESS graph visually demonstrates the difference in the dose-response of deterioration for each lactate level between groups, generally being more strongly associated with the outcomes in patients with infection than in those without infection. Yet, despite the differences in lactate production and metabolism, the association between lactate concentrations and deterioration was strong and added prognostic value in both groups.

Prior studies have established the clinical utility of using lactate concentration in patients with a variety of critical illnesses [2,5,6,11–14,19]. For instance, Shapiro et al. showed that in ED patients with infection, the 28-day in hospital mortality rate was 28% if a single lactate was > 4 mmol/L, 9% if it was 2.5 to 4, and 4.9% if lactate levels were normal.^2^ Our results are consistent with these prior studies, demonstrating the prognostic ability of lactate measurements when predicting adverse outcomes. Yet these studies are generally limited to a single disease and do not allow a comparison of a serum lactate’s prognostic value between different disease categories.

Our study differs from most prior investigations by enrolling an undifferentiated patient population, allowing the association between lactate concentrations and adverse outcomes in patients with and without infection to be evaluated side by side. This analysis, stratified by the apparent presence of infection, supports the conclusion that the relationship between serum lactate measurements and adverse outcomes is not limited to a specific disease. This finding is consistent with a recently published report by Haidl et al.,^16^ which demonstrated that serum lactate levels confer an increased risk of 10-day mortality among undifferentiated patients who present to the ED. Our study also furthers the Haidl et al. findings by assessing for differences in the predictive value of lactate levels based on the underlying disease category. Our stratified analysis suggests qualitatively that lactate levels have a similar degree of association with deterioration in patients with and without infection. Furthermore, while a difference in the association between lactate concentrations and adverse outcomes likely exists in between infectious and non-infectious diseases, best seen in the LOESS graph (Figure 2a+b), adding lactate > 4mmol/L to the best clinical models in both patient groups, added value to the prediction of adverse outcomes. These data support the clinical use and similar interpretation of lactate concentrations in ED patients with and without infection when predicting adverse outcomes.

When considering the secondary outcome of mortality, our study does contrast with the study by del Portal et al., which found that in an undifferentiated ED population of patients > 65 years old initial lactate levels were associated with increased mortality in both sepsis and non-sepsis patient populations. In part, the inability of our study to show that lactate added value to the prediction of mortality in patients without infection can be explained by differences between the studied populations. The population studied by del Portal et al. was older with a mean age of 77.2 (±7.8) years.

Also, this study used patients from 2004–2006, when lactate levels were less frequently ordered, especially for patients without infection. Our study includes a more recent patient population, which more closely reflects the current utilization of lactate levels in patients with AVS. However, similar to our analysis, the prediction model used by del Portal performed better in patients with infection than in the non-infected patient population.^8^ Furthermore, our study was not powered to identify a difference in mortality, and it is possible that a difference may have been detected with a larger sample size.

### Future Directions

This study creates a foundation for further investigation into the relationship between lactate levels and outcomes in patients with and without infection. Lactate clearance is also being studied across the spectrum of disease to predict outcomes. A study similar to this analysis comparing the prognostic value of lactate clearance in a cohort including both infected and non-infected patients is warranted.

## LIMITATIONS

This study has a number of limitations. Identifying a broad group of patients who were critically ill required us to screen using vital sign criteria that can be caused from less urgent etiologies. Our vital sign thresholds allowed high sensitivity for critical illness, yet identified many patients who were not critically ill. The excluded diagnoses were decided a priori to represent a very low-risk group that would require minimal stabilizing interventions, and they account for the majority of excluded patients. While these patients were excluded prior to the current analysis, it is reasonable to expect that some of these patients would have a serum lactate measured during clinical care. Other comorbidities (i.e., liver disease) and medications (i.e., metformin) can affect the lactate level, yet may not be related to the acute illness treated in the ED. This study does not account for these alternative factors influencing lactate levels, as an ED clinician would do in a real clinical setting. Lactate concentrations should be interpreted with discretion when non-acute factors that may influence the level are present.

As an observational study, the physician’s decision to obtain lactate measurements is likely to introduce selection bias. However, in our institution it is common to obtain a lactate value in patients with signs of critical illness regardless of the underlying cause. Therefore, our vital sign thresholds likely reduce the degree of selection bias present based on physician ordering. Still, many patients were excluded because lactate measurements did not occur in the ED, and we do not know the rate of deterioration in this group.

The outcomes we chose for our composite outcome of deterioration are not all encompassing. Other investigators may have included more outcomes, the need for non-invasive ventilator support. While this approach likely decreased the number of composite outcomes in our study, we believe that using acute renal failure, vasopressor administration, intubation and mortality, created a composite outcome that clearly represents significant clinical events.

Misclassification of patients is another potential limitation, although using a second reviewer to assess agreement decreases this likelihood. Our kappa of 0.85 was fairly strong, yet some disagreements did occur, for instance, when considering whether bacterial translocation may have occurred in a small bowel obstruction or whether a COPD exacerbation was triggered by a respiratory infection. The PI determined the final diagnosis from the medical record, which may include only limited data to determine a diagnosis, thus contributing to misclassification bias. This fact would most likely not influence the study results, since it is unlikely to be systematically related to a patient’s lactate level. Therefore, such misclassification would likely weaken the apparent relationships between lactate levels and outcomes. Lastly the treating clinicians were not blinded to results of lactate analysis and we do not know how this information may have affected clinical care, and thereby possibly the outcome parameters (i.e., use of vasopressors). This could have an impact on the ability to investigate lactate as a predictor of this outcome. However, within our ED the decision to use vasopressors is based on blood pressure parameters, not guided by lactate levels.

## CONCLUSION

Lactate levels measured in ED patients exhibiting AVS correspond with adverse outcomes during their hospitalization in the presence and absence of infection. While differences in the predictive value may exist between patients with and without infection, lactate concentrations do add prognostic value in both groups at similar levels, justifying the utilization and similar interpretation of lactate levels regardless of underlying disease.

## Figures and Tables

**Table 1:** Population characteristics of emergency department patients with abnormal vital signs in a study analyzing serum lactate levels as a measure of adverse outcomes.

Variable	Without infection (n=202)	With infection (n=286)	aP-value
Age (median ±SD)	62 ±18	66 ±18	0.95
Female (n, %)	100 (49.5%)	141 (49.3%)	0.97
Past medical history (n, %)			
Diabetes	69 (34.2%)	69 (24.1%)	0.02
Coronary artery disease	39 (19.3%)	51 (17.8%)	0.68
Myocardial infarction	14 (6.9%)	12 (4.2%)	0.19
Congestive heart failure	51 (25.2%)	49 (17.1%)	0.03
Hypertension	101 (50%)	129 (45.1%)	0.29
Dementia	12 (5.9%)	23 (8%)	0.38
Active cancer	47 (23.3%)	72 (25.2%)	0.63
Chronic obstructive pulmonary disease	37 (18.3%)	53 (18.5%)	0.95
Liver disease	17 (8.4%)	17 (5.9%)	0.29
Chronic renal insufficiency	13 (6.4%)	20 (7%)	0.81
Dialysis	19 (9.4%)	20 (7%)	0.33
History of stroke	11 (5.4%)	19 (6.6%)	0.59
Vital signs (median ±SD)			
Heart rate	104 ±24	110 ±24	0.02
Temperature	98.0 ±1.3	98.8 ±2.3	<0.001
Systolic blood pressure	105 ±30	102 ±28	0.5
Diastolic blood pressure	64 ±19	60 ±17	0.14
Respiration rate	20 ±6	20 ±5	0.89
SO2 (%)	97±4	98 ±4	0.07

a Statistical test used: Continuous variables: Student’s t-test.

Categorical variables: Chi-squared test.

**Table 2:** Distribution of deterioration by stratified lactate value for both infected and non-infected patients.

	Without infection (n, %)	Infection (n, %)
Lactate < 2.5 (n=342)	142 (70.3)	200 (69.9)
2.5 ≤ Lactate ≤ 4 (n=100)	40 (19.8)	60 (21.0)
Lactate > 4 (n=46)	20 (9.9)	26 (9.1)

**Table 3:** Outcome measures in the population of ED patients.

	Without infection (n=202)	With infection (n=286)	aP-value
Length of stay (days, median, IQR^b^)	4 (2–7)	5 (3–8)	0.03
Deterioration^c^ (n, %)	68 (33.7)	100 (35)	0.76
Acute renal failure (n, %)	32 (15.8)	46 (16.1)	0.94
Intubation (n, %)	27 (13.4)	38 (13.3)	0.98
Vasopressors during hospitalization (n, %)	23 (11.4)	69 (24.1)	<0.001
Death (n, %)	25 (12.4)	36 (12.6)	0.95
Shock in ED (n, %)	33 (16.3)	81 (28.3)	0.002

a Statistical test used for variable length of stay: Mann Witney, categorical variables: Chi-squared test

b IQR: Interquartile range

c Deterioration was considered to be one or more of the following outcomes during hospitalization: acute renal failure, non-elective intubation, vasopressors requirement, death.

**Table 4:** Multivariable logistic regression models.

Variable	AOR^a^	95% CI	P-value
a: For deterioration in patients with infection			
Lactate > 4	4.84	1.66–14.13	0.004
Systolic blood pressure < 90 mmHg	2.48	1.32–4.66	0.005
Triage acuity^b^	0.44	0.28–0.68	<0.001
Blood urea nitrogen	1.05	1.03–1.08	<0.001
b: For mortality in patients with infection			
Lactate > 4	4.41	1.7–11.45	0.002
History of stroke	4.52	1.42–14.33	0.01
Blood urea nitrogen	1.02	1.00–1.04	0.03
Triage acuity^b^	0.26	0.12–0.59	0.001
c: For deterioration in patients without infection			
Lactate > 4	3.6	1.25–10.32	0.02
Triage acuity^b^	0.49	0.29–0.82	0.007
History of stroke	0.11	0.01–1.11	0.06
Blood urea nitrogen	1.02	1.01–1.03	0.002
Altered mental status	5.9	1.89–18.4	0.002
d: For mortality in patients without infection			
Lactate > 4	1.19	0.27–5.21	0.81
Age	1.04	1.01–1.07	0.01
Active cancer	3.09	1.43–15.02	0.01
Altered mental status	4.63	1.43–8.13	0.02

a Adjusted odds ratio;

b Triage acuity determined by emergency department nurse
